# Hybrid Diagnostic Framework for Interpretable Bearing Fault Classification Using CNN and Dual-Stage Feature Selection

**DOI:** 10.3390/s25206386

**Published:** 2025-10-16

**Authors:** Mohamed Elhachemi Saouli, Mostefa Mohamed Touba, Adel Boudiaf

**Affiliations:** 1 LESIA Laboratory of Research, University of Mohamed Khider Biskra, Biskra 07000, Algeria; 2 Li3CUB Laboratory of Research, University of Mohamed Khider Biskra, Biskra 07000, Algeria; mostefa.touba@univ-biskra.dz; 3 Research Center in Industrial Technologies—CRTI, Cheraga, P.O. Box 64, Algiers 16014, Algeria; a.boudiaf@crti.dz

**Keywords:** fault diagnosis, rotary machinery, CNN transfer learning, dual-stage feature selection, supervised classification, interpretability

## Abstract

Timely and accurate fault diagnosis in rotary machinery is essential for ensuring system reliability and minimizing unplanned downtime. While deep learning approaches, particularly Convolutional Neural Networks (CNNs), have demonstrated strong performance in vibration-based fault classification, their limited interpretability poses challenges for adoption in safety-critical environments. To address this, the present study introduces a hybrid diagnostic framework that integrates CNN-based transfer learning with interpretable supervised classification, aiming to enhance both predictive accuracy and model transparency. A key innovation of this work lies in the dual-stage feature selection process, combining Analysis of Variance (ANOVA) and Permutation Feature Importance (PFI) to refine deep features extracted from a pre-trained VGG19 network. This strategy improves both dimensionality reduction and classification performance in a statistically grounded, model-agnostic manner. Furthermore, SHapley Additive exPlanations (SHAP) are employed to interpret the predictions, offering insight into the most influential features driving the classification decisions. Experimental evaluation on the Case Western Reserve University (CWRU) bearing dataset confirms the effectiveness of the proposed approach, achieving 100% classification accuracy using ten-fold cross-validation. By uniting high performance with transparent decision-making, the framework demonstrates strong potential for explainable and reliable fault diagnosis in industrial settings.

## 1. Introduction

The accurate and timely diagnosis of failures in rotary machinery remains a critical objective in industrial maintenance and operational efficiency. Among various diagnostic techniques, vibration analysis has emerged as one of the most effective due to its high sensitivity to structural and mechanical anomalies [1,2]. The Fourth Industrial Revolution has driven the integration of vibration analysis with artificial intelligence (AI) to support predictive maintenance and improve system reliability and performance [3]. However, this integration also introduces several new challenges, including data noise [4], complex feature extraction [5], high dimensionality [6], limited model interpretability [7], and the necessity for real-time processing [8]—factors that collectively complicate the development of accurate and dependable bearing fault diagnosis systems.

Early efforts predominantly employed machine learning (ML) models that relied on handcrafted feature extraction from vibration signals. Common approaches included time-domain descriptors such as root mean square (RMS), mean, kurtosis, and skewness [9]; frequency-domain methods such as Fourier Transform (FT) [10], Envelope Analysis (EA) [11]; and time–frequency analyses such as Short-Time Fourier Transform (STFT) [12], Hilbert–Huang Transform (HHT) [13], Wavelet Analysis (WA) [14], and Variational Mode Decomposition (VMD) [15]. These features were fed into classifiers to detect specific faults. While effective, such methods rely heavily on domain expertise, extensive preprocessing, and manual feature engineering [16]. Additionally, ML classifiers may underperform when faced with high-dimensional or noisy input features that are not sufficiently informative [17,18].

Beyond traditional approaches, recent developments have introduced data-driven methods such as phase diagram analysis, which extract features directly from the geometric structure of signals in reconstructed phase space, without requiring prior knowledge or extensive preprocessing [19]. These techniques have shown strong potential in capturing transient and nonlinear dynamics, thereby enhancing feature extraction for machinery fault diagnosis. However, their effectiveness can be constrained by noise sensitivity, computational complexity, and challenges in parameter selection.

To address these limitations, deep learning (DL) particularly using Convolutional Neural Networks (CNNs) has emerged as a powerful alternative. CNNs automatically learn hierarchical features from raw data, enabling end-to-end diagnostics. Transfer learning (TL) with pre-trained CNN models enhances efficiency by leveraging knowledge from large-scale datasets, thereby reducing the need for extensive labeled data and computational resources [20].

Despite their strong performance, CNNs are often criticized for their opaque internal representations. This limits their deployment in safety-critical settings, where decision transparency is essential. While feature visualization techniques provide some insight into CNN behavior, practical interpretability often remains elusive especially when decisions are based on complex feature hierarchies [21].

In parallel, feature selection has emerged as an important strategy to reduce dimensionality, improve learning efficiency, and enhance model transparency. Methods such as filter [22], wrapper [23], and embedded [24] approaches aim to identify the most informative features, thus facilitating better generalization and interpretability. However, when applied in isolation, these methods are still limited by the quality and nature of the input features [23,25].

To overcome these limitations, hybrid frameworks that combine deep feature extraction with traditional ML classification have been proposed. These approaches integrate various combinations of signal processing, CNN-based feature learning, and conventional classifiers. For example, a compressive sensing (CS)-based filter feature selection approach was proposed in [22], applied to features extracted using Fast Fourier Transform (FFT) and wavelet transform, with classification performed using Artificial Neural Networks (ANN) and Support Vector Machines (SVM). Another study introduced an ensemble learning framework based on transfer learning with a LeNet-5 model, where features extracted from wavelet-transformed signals were classified using a Random Forest (RF) algorithm [26]. Similarly, a three-stage model in [27] utilized time–frequency-domain feature extraction, followed by Neighborhood Component Analysis (NCA) for selection and classification via ML algorithms. Additional frameworks have explored combinations such as CNN-SVM [28], VMD-ANN [29], and Wavelet-CNN [30]. While many of these methods improve diagnostic accuracy, relatively few explicitly address the interpretability of the diagnostic process particularly at the interface between deep learning feature extraction and decision-level classification.

Most existing fault diagnosis models focus primarily on improving accuracy, with limited emphasis on understanding or explaining model decisions. As a result, the integration of deep feature learning with interpretable supervised classification remains an underexplored yet critical area for developing trustworthy diagnostic systems.

In light of these considerations, this study proposes a hybrid interpretable diagnostic framework that balances predictive performance with interpretability. The proposed approach combines CNN-based VGG19 transfer learning for feature extraction with a dual-stage feature selection mechanism, leveraging Analysis of Variance (ANOVA) and Permutation Feature Importance (PFI) to identify the most informative features. These refined features are then used to train multiple supervised learning algorithms, including Random Forest (RF), Extreme Gradient Boosting (XGBoost), Logistic Regression (LR), K-Nearest Neighbors (KNN), Artificial Neural Networks (ANN), and Support Vector Machines (SVM). This classification is followed by models’ explanation via SHAP (SHapley Additive exPlanations) analysis. This combination contributes to building a high performance and interpretable intelligent bearing fault diagnosis.

While the internal representations learned by CNNs remain partially opaque, this study improves interpretability by analyzing the CNNs’ feature outputs. Through systematic selection and analysis of these deep features, and their use in interpretable supervised models, the framework enables a clearer understanding of which characteristics contribute most significantly to fault classification. This enhances diagnostic transparency without sacrificing accuracy.

The proposed framework is validated using the Case Western Reserve University (CWRU) bearing dataset. The results demonstrate high classification performance results, achieving 100% accuracy. The comparative results show the enhancement in accuracy, adopting the dual-stage feature selection approach. Comparative analysis with existing techniques confirms the effectiveness and reliability of the proposed method. This paper mainly contributes to the following:

- Proposes a dual-stage approach for dimensionality reduction and feature selection based on ANOVA and PFI, designed for general applicability across various supervised machine learning models.
- Investigates the interpretability of the CNN black-box by analyzing the extracted deep features.
- Provides ML model explanation.
- Improves both classification performance and interpretability of intelligent bearing fault diagnosis framework.

The remainder of this paper is structured as follows: Section 1 is an introduction. Section 2 presents the bearing fault and vibration analysis in rotary machinery. Section 3 illustrates a review of the dataset used in this study. Section 4 introduces the feature extraction technique. Section 5 describes the proposed dual-stage feature selection approach. Section 6 outlines the overall methodology. Section 7 presents the experimental results, including a comparative study. Section 8 discusses the model interpretability using SHAP analysis, and finally, the paper conclusion in Section 9.

## 2. Bearing Faults and Vibration Analysis in Rotary Machines

Electric motors, as a central class of rotary machinery, are responsible for nearly half of the world’s total electricity usage and account for around 70% of industrial energy demand [31]. This extensive reliance highlights their critical importance in maintaining the continuity and efficiency of modern industrial processes. Electrical motors, in particular, are expected to operate continuously over their intended service life while avoiding unexpected shutdowns or severe malfunctions. However, despite their structural robustness and proven reliability, studies have shown that these machines are still prone to progressive wear and failure. Statistical investigations conducted by the Institute of Electrical and Electronics Engineers (IEEE) [32] reveal that bearing faults are implicated in approximately 40–50% of motor failures (Table 1), thereby establishing bearings as the single most common and severe source of malfunction in rotary machinery.

Several condition monitoring techniques have been developed to reduce failure risks and enable early fault detection.

The methods include the following:

- Infrared Thermography (IT);
- Ultrasonic Testing (UT);
- Electrical Signature Analysis (ESA);
- Acoustic sound-based condition monitoring (ASCM).

These methods provide useful diagnostic information by identifying thermal anomalies, high-frequency acoustic emissions, current irregularities, or abnormal noise patterns, respectively [33]. However, their effectiveness in detecting incipient mechanical faults is often limited, and their reliability may be affected by operational and environmental conditions.

By contrast, Vibration Analysis (VA) has become the most reliable and widely applied approach for bearing fault diagnosis. Localized defects in the inner race, outer race, or rolling elements generate periodic impact responses that manifest as distinct vibration signatures, enabling accurate fault localization and classification. The method offers comprehensive diagnostic information across the time, frequency, and time-frequency domains, while remaining non-intrusive, cost-effective, and easily deployable using standard accelerometers, thus supporting predictive maintenance and large-scale industrial monitoring.

## 3. Dataset Review

**Figure 1:**
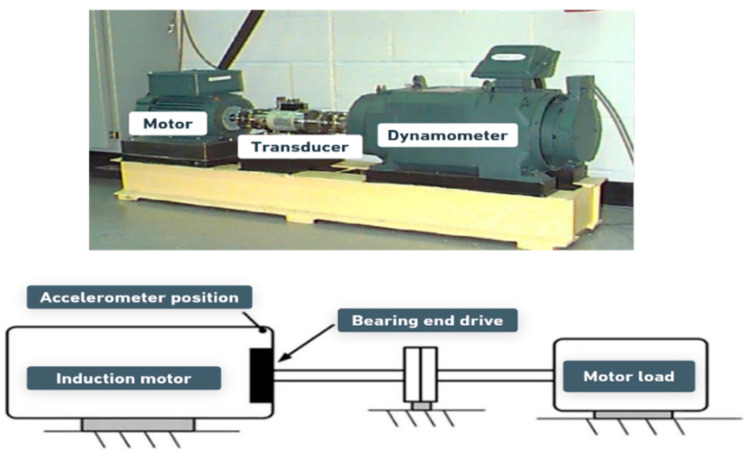
The CRWU benchmark.

This study uses the vibration signals obtained from the Case Western Reserve University (CWRU) Bearing Data Centre [34]. The public accessibility of this dataset facilitates fair and consistent comparisons of various methodologies proposed for bearing fault diagnosis, thereby serving as both a benchmark and a source of motivation for this research. The CWRU experimental benchmark for the data collection shown in Figure 1 consists of an electric induction motor on one side, a torque transducer in the middle, and a dynamometer on the other end to simulate the load. The experimental data was obtained based on the accelerations generated by an electric motor under various conditions, including altering the nature of failure, severity, and the shaft rotational speed.

**Figure 2:**
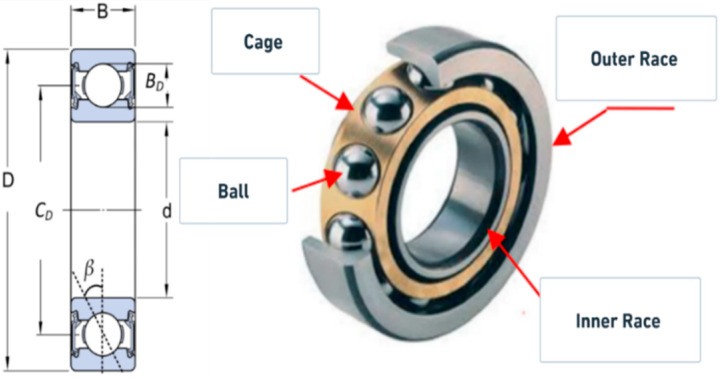
Bearing’s components.

The bearing used in this study is the SKF 6205-2RSL JEM single-row deep groove ball bearing, which consists of four main components: the inner race, outer race, ball, and cage. These components are illustrated in Figure 2, while the detailed technical specifications of the bearing are provided in Table 2.

The bearing defects can be caused by different reasons, including insufficient lubrication, mechanical friction, rotor misalignment [2]. Accordingly, four types of bearing faults are defined: (the inner race fault (*f_IRF_*), the outer race fault (*f_ORF_*), the ball fault (*f_BF_*), and the cage fault (*f_CF_*)). Each of them has a distinctive signature, known as characteristic frequency. The associated frequencies for each failure in function of rotation frequency (*fr*) of bearing are calculated using the following equations [35]:

(1) $$f_{IRF}=\frac{N}{2}f_{r}\left(1+\frac{B_{D}}{C_{D}}\cos\beta \right)$$

(2) $$f_{ORF}=\frac{N}{2}f_{r}\left(1-\frac{B_{D}}{C_{D}}\cos\beta \right)$$

(3) $$f_{BF}=\frac{C_{D}}{B_{D}}f_{r}\left(1-\frac{{B_{D}}^{2}}{{C_{D}}^{2}}\cos^{2}\beta \right)$$

(4) $$f_{CF}=\frac{f_{r}}{2}\left(1-\frac{B_{D}}{C_{D}}\cos\beta \right)$$

## 4. Feature Extraction

Effective classification of bearing faults essentially depends on the ability to identify and leverage the most relevant features. Therefore, feature extraction is considered the most critical and sensitive step in the classification task, as it directly influences the accurate fault recognition and distinction. In this work, a CNN is employed to extract features from two-dimensional 2D image representation of vibration signal, based on transfer learning using the pre-trained model VGG19. A brief description of the pre-trained model is provided in the next section:

### 4.1. Signal Conversion

**Figure 3:**
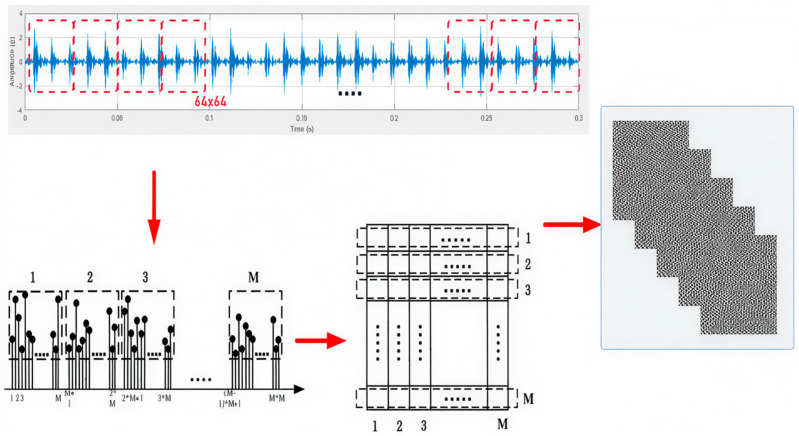
Converting 1D vibration signal to 2D image methodology.

The concept behind signal-to-image conversion, as illustrated in Figure 3, involves transforming raw time-domain signals into images, offering an alternative preprocessing approach. To create an M × M image, a segmented signal of length $M^{2}$ is derived from the raw vibration signal. In this study, 64 × 64 grayscale images are generated, with the segmented signal having a length of 4096. The segmented signal values are represented as L(i), where i = 1, …, M^2^, while the pixel strength of the resulting image is denoted by P (j, k), where j, k = 1, …, M. The converted grayscale images are given by the following formula:

(5) $$P\left(j,k\right)=round\;\left\{\frac{L\left(\left(j-1\right)\times M+K\right)-\min\left(L\right)}{\max\left(L\right)-\min\left(L\right)}\;\times 255\right\}$$

Here, round {⋅} represents the rounding function, and the transformation maps values to the interval [0, 255], producing a grayscale image [36]. This straightforward mapping converts 1D raw signals into 2D textured representations, where each operational condition whether normal or indicative of a bearing defect corresponds directly to a distinct class of similarly textured images. Notably, this approach does not rely on any predefined parameters, making it a flexible and effective preprocessing technique. The choice of this specific 1D to 2D conversion method over other alternatives, such as wavelet scalograms, was guided by its simplicity and computational efficiency. Unlike scalograms, which require careful selection of mother wavelets and scale parameters potentially introducing additional complexity and computational overhead the proposed method is parameter-free and preserves the temporal texture of the signal directly. This enables effective feature representation while allowing for direct application of image-based deep learning techniques, such as convolutional neural networks, without prior transformation design.

Furthermore, the selection of a 64 × 64 image size, corresponding to a segment length of 4096, was based on practical considerations. This configuration strikes a balance between capturing sufficient signal detail and maintaining computational efficiency. A segment length of 4096 provides enough data to preserve fault-related characteristics, while the 64 × 64 image format ensures that the resulting images are neither too large to hinder model training nor too small to lose critical information. This resolution is also consistent with prior literature, where similar image sizes have proven effective in signal-based condition monitoring tasks.

### 4.2. Feature Extraction Using VGG19

**Figure 4:**
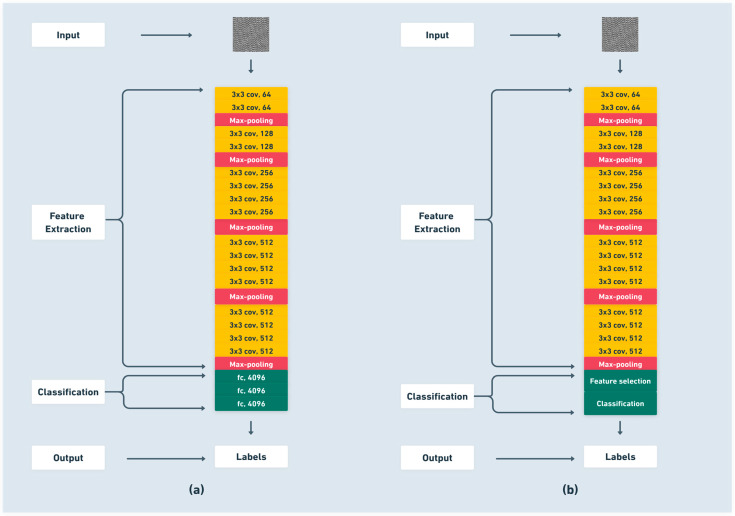
(**a**) VGG19 pre-trained model. (**b**) The proposed ensemble of VGG19 deep feature extraction, dual-stage feature selection and machine learning classification.

VGG19, considered one of the most frequently used paradigms in image classification, is a deep learning architecture designed on a convolutional neural network, with nineteen layers, sixteen of which are convolutional, while the remaining three are fully connected layers. Its design stands on 3 by 3 filters that allow the model to catch the most relevant details. VGG19 uses the ImageNet dataset. This model is designed to classify images into one of a thousand distinct object categories [37]. As depicted in the architecture shown in Figure 4a, the first 16 layers consist exclusively of convolutional operations used for feature extraction. These convolutional layers are organized into five distinct blocks, each followed by a max-pooling layer to progressively reduce spatial dimensions while preserving essential features. The remaining layers constitute the classification component of the network. The model input consists of images resized to 224 × 224 resolution with three color channels, and the output corresponds to image labels. In this study, the original 64 × 64 grayscale images derived from vibration signals are resized to 224 × 224 and replicated across three channels prior to input, ensuring compatibility with VGG19 without compromising signal information.

VGG19 was selected over other pre-trained CNN architectures primarily due to its straightforward and well-established structure, which facilitates the visualization and understanding learned features, an important consideration in condition monitoring applications. While deeper networks like ResNet offer improved accuracy via residual connections, their increased complexity can hinder transparent analysis of feature extraction. The simplicity of VGG19 thus enables effective feature representation alongside easier interpretability.

In this study, we use the pre-trained VGG19 model to extract features [37]. However, several machine-learning techniques are applied for classification. This deep feature extraction process generates a huge number of features, some of which are impactful, while others are not. This issue results in increased computational time due to an oversized feature vector, along with misclassification problems caused by irrelevant features and high dimensionality. Therefore, there is a need for feature selection methods to reduce the number of features by choosing only the most important ones as mentioned in Figure 4b. The feature selection in our case is performed using combined feature selection methods.

## 5. Feature Selection

In the bearing fault diagnosis, the extraction and interpretation of meaningful features from complex models is of paramount importance [38]. The proposed work leverages deep features extracted via VGG19, a pre-trained CNN, to enhance the diagnostic capability of ML algorithms. While deep learning models such as the VGG19 have demonstrated superior performance in feature extraction of vibration signal [39,40], they inherently function as ‘’black box’’ models [7,8,9,10,11,12,13,14,15,16,17,18,19,20,21,22,23,24,25,26,27,28,29,30,31,32,33,34,35,36,37,38,39,40,41]. This characteristic impedes interpretability, which is crucial in critical applications such as fault detection in rotating machinery. Consequently, understanding the relevance and impact of deep features becomes essential, not only to improve ML model performance but also to foster trust, transparency, and insight into model behavior.

To address this interpretability challenge and simultaneously enhance model efficiency, a hybrid feature selection methodology is proposed, combining Analysis of Variance (ANOVA) F-statistics with Permutation Feature Importance (PFI). This dual-stage approach capitalizes on the complementary strengths of both techniques to reduce feature dimensionality, eliminate irrelevant or redundant features, and provide insights into the relationships between features and model predictions.

### 5.1. ANOVA-Based Pre-Filtering

The first stage of the proposed method utilizes the ANOVA F-statistic as a preliminary filter to assess the statistical significance of each extracted feature with respect to the target fault classes. ANOVA, a well-established parametric statistical test, evaluates the variance between group means relative to the variance within the groups, thereby identifying features that contribute significantly to class separation. Mathematically, the F-statistic is defined as follows [42]:

(6) $$F=\frac{Variance\;between\;group}{Variance\;within\;group}=\frac{\sum_{i=1}^{k}{n_{i}\left({\bar{x}}_{i}-\bar{x}\right)}^{2}/(K-1)}{\sum_{i=1}^{k}\sum_{j=1}^{n_{i}}{n_{i}\left({x_{ij}-\bar{x}}_{i}\right)}^{2}/(N-k)}$$

where ${\bar{x}}_{i}$ is the mean of group i, $\bar{x}$ is the overall mean, $x_{ij}$ denotes the j-th observation in group i, $n_{i}$ is the number of samples in group i, k is the number of groups, and N is the total number of samples. Features with low F-values, indicating minimal discriminatory power, are discarded, thereby reducing dimensionality and improving computational efficiency.

The rationale for selecting ANOVA lies in its simplicity, scalability, and effectiveness in identifying linearly separable features [43]. Importantly, it helps to mitigate the adverse effect of multicollinearity, by removing highly correlated or statistically insignificant features in advance [44].

### 5.2. Permutation Feature Importance: Model-Agnostic Interpretability

Following the ANOVA filtering, the retained subset of features undergoes evaluation using Permutation Feature Importance. PFI is a model-agnostic technique that quantifies the importance of each feature by measuring the change in the model’s prediction error upon randomly shuffling its values [45]. The greater the degradation in performance, the more important the feature is deemed to be.

The key advantages of PFI include the following [45,46,47]:

- Applicability to any machine learning model, including tree-based, kernel-based, and neural models.
- Effectiveness in detecting nonlinear relationships and complex feature interactions.
- Enhanced interpretability by directly linking features to model output changes.

### 5.3. Implementation Steps

ANOVA is selected for its simplicity, scalability, and effectiveness in identifying linearly separable features, while also addressing multicollinearity, an inherent limitation of Permutation Feature Importance (PFI). As PFI is sensitive to highly correlated features, which may distort the interpretation of feature relevance, the preliminary application of ANOVA effectively reduces redundancy and filters out statistically insignificant features. This integration enhances the stability and reliability of the importance rankings generated by PFI, ultimately supporting a more interpretable and robust feature selection process.

**Figure 5:**
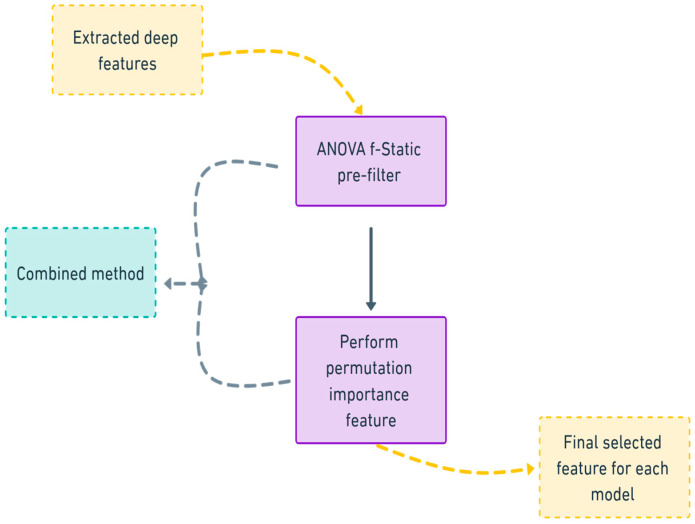
Workflow of the adopted dual-stage feature selection method.

The combined feature selection approach involves the following steps as illustrated in workflow in Figure 5:

1. Feature extraction: Extract deep features from the penultimate layer of the VGG19 model for each input sample.
2. Preform ANOVA F-test filtering:
  - Compute the F-statistic for each feature across different fault classes.
  - Select features exceeding a predefined significance threshold.
3. Preform Permutation Feature Importance:
  - Apply the selected features to train a machine learning model.
  - Iteratively permute each feature and measure the impact on model accuracy.
  - Rank features based on the observed degradation in performance.
4. Final selected feature subset: Retain top-ranked features for subsequent model training and evaluation.

### 5.4. Benefits of the Combined Approach

The integration of ANOVA and PFI offers a robust framework for both dimensionality reduction and interpretability enhancement. From a practical standpoint, this combined methodology contributes to:

Improved interpretability: By ranking and analyzing the contribution of individual deep features, it provides insights into the inner workings of the VGG19-based feature extractor, helping demystify its ‘‘black box’’ nature.

Enhanced model performance: By eliminating irrelevant and redundant features, the resulting ML models demonstrate increased accuracy and reduced generalization error.

Reduced execution time: The pruning of high-dimensional feature sets streamlines training and inference, making the approach suitable for real-time or embedded diagnostic applications.

Model behavior understanding: Facilitates a deeper understanding of how specific features influence the classifier’s decision boundaries, thereby aligning ML predictions with domain knowledge in fault diagnostics.

In summary, the proposed dual-stage feature selection framework serves as a critical enabler for both the performance and interpretability of machine learning models in bearing fault diagnosis. By bridging the gap between deep feature representations and transparent decision-making, this approach enhances the reliability and applicability of intelligent the bearing diagnostic systems in industrial settings.

## 6. Proposed Methodology

**Figure 6:**
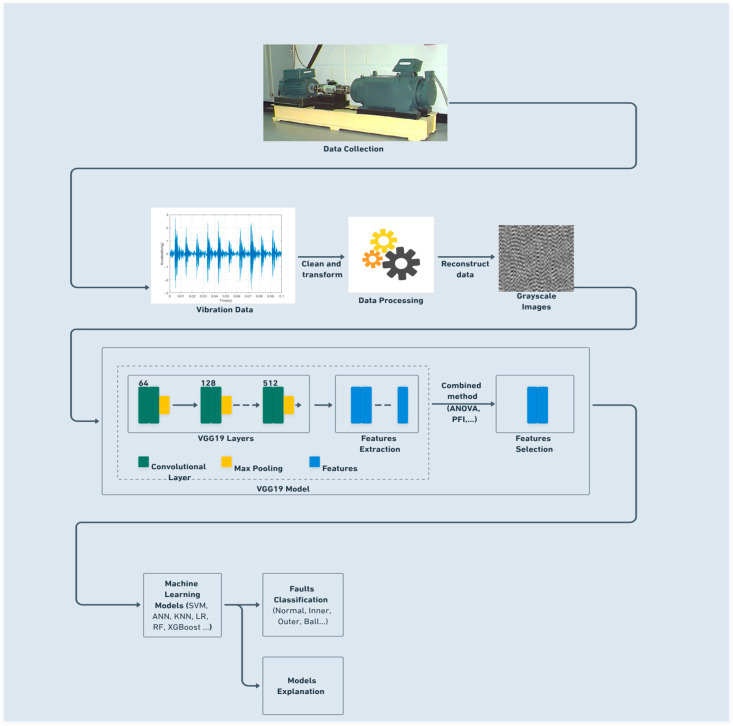
Workflow for the proposed strategy.

The workflow of the proposed methodology, given in Figure 6, intends to improve the performance of different machine learning algorithms in classifying rolling bearing faults. The conducted experiment using the (CWRU) dataset analyzed the effectiveness of the bearing fault diagnosis system. The proposed strategy is based on integration between deeply extracted features obtained from deep CNN (VGG19) transfer learning, dual-stage feature selection approach using the ANOVA algorithm and PFI and SHAP analysis for supervised classification model explanation. The proposed system works in five main steps as follows:

- Converting the vibration signal to a 64 by 64 grayscale image.
- Extracting the deep features using the VGG19 pre-trained model.
- Selecting the most important features using dual stage feature selection.
- Classifying by different machine learning classifiers.
- Explain models using SHAP analysis.

Within the first step, the vibration signal is converted to a 64 by 64 grayscale image. At first, the collected vibration signals are subjected to a data preprocessing process to prepare them for conversion. The process consists of removing errors from the raw data, eliminating duplicate data, and transferring the data type. The second step is about feature extraction. VGG19 extracts all deep features, generating 4096 features from the output of the first fully connected layer (fc1) of the pre-trained VGG19 model. The next move is more sensitive, the third step in our proposed strategy. It concerns feature selection. In this step, features are selected, employing the dual stage approach, to use them in a fourth move, involving classification utilizing various machine learning classifiers. The last step in our methodology workflow is dedicated to explaining the outcomes of the fourth step.

## 7. Results and Discussion

**Figure 7:**
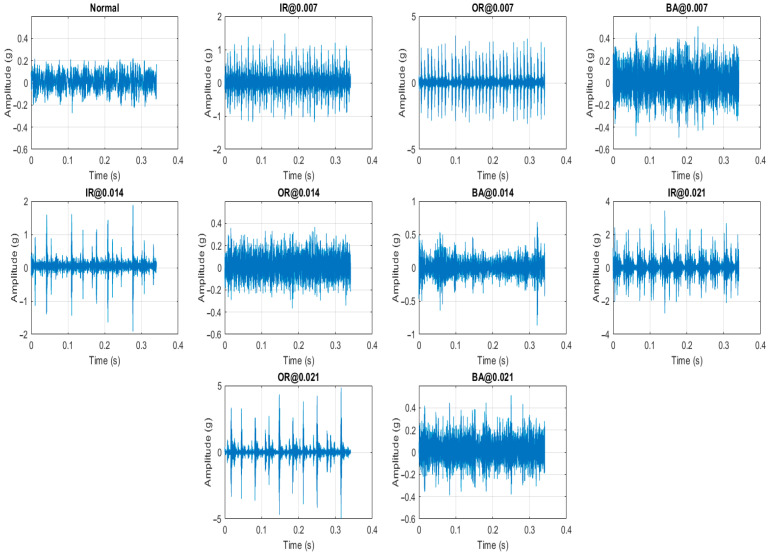
Samples of raw vibration signals under different operating conditions form CRWU dataset.

The vibration data were captured by an accelerometer fixed to the bearing housing at the drive end of the induction motor and collected under various conditions, including health operation, inner race fault, outer race fault, and ball fault, at different motor loads of 0, 1, 2, and 3 hp and for fault diameters of 0.007, 0.014 and 0.021 inches. The data are recorded at a sampling rate of 12,000 samples per second and saved in MATLAB format files. Samples of the vibration signals under the different operating conditions are shown in Figure 7.

**Figure 8:**
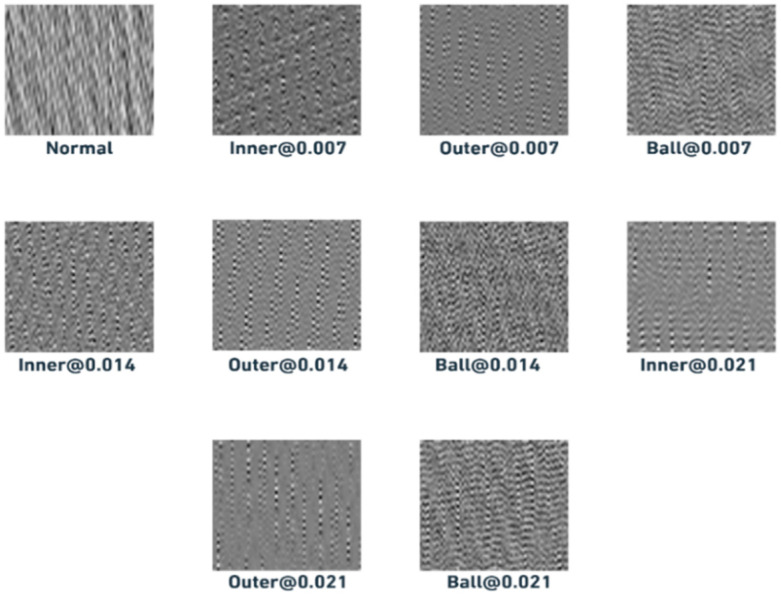
Samples of the converted greyscale image signals under different operating conditions.

**Figure 9:**
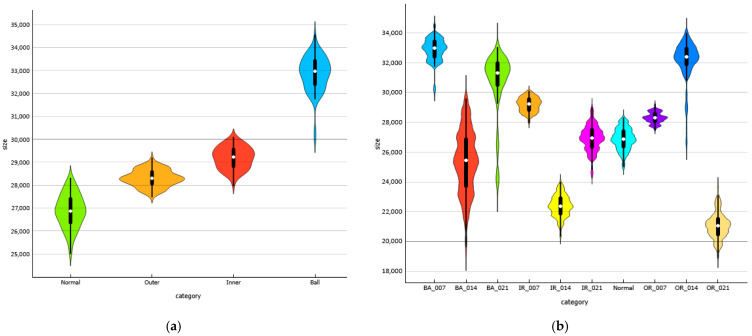
Violin plot: (**a**)—distribution of Dataset A, (**b**)—distribution of Dataset B.

Table 3 describes the ten different faulted bearing classes used within this experimental dataset. In this experiment, we construct two datasets, each one containing different classes of rolling bearing faults. Dataset A has four classes corresponding to the first four labels of Table 3, and dataset B has ten classes covering all bearing labels in Table 3. The final construction of datasets is presented in Table 4. Figure 8 presents samples of reconstructed images representing the used classes. The datasets are balanced, and each category has the same number of images. Figure 9 represents a violin plot of both dataset A and B, it shows a detailed view of the dataset’s distribution.

Following the extraction of deep features using the pre-trained VGG19, a two-stage feature selection strategy is employed to identify the most relevant and informative features for each classifier from deep extracted features. In the first stage, ANOVA is utilized to statistically rank all extracted features based on their ability to discriminate between fault classes. The top-ranked features are then selected to reduce dimensionality while retaining the most diagnostically significant information.

In the second stage, PFI is applied independently for each supervised learning classifier. This step quantifies the contribution of each selected feature to the model’s predictive performance by measuring the decrease in accuracy when a feature’s values are randomly shuffled. This classifier-specific refinement ensures optimal feature relevance tailored to each model’s structure and learning dynamics.

**Figure 10:**
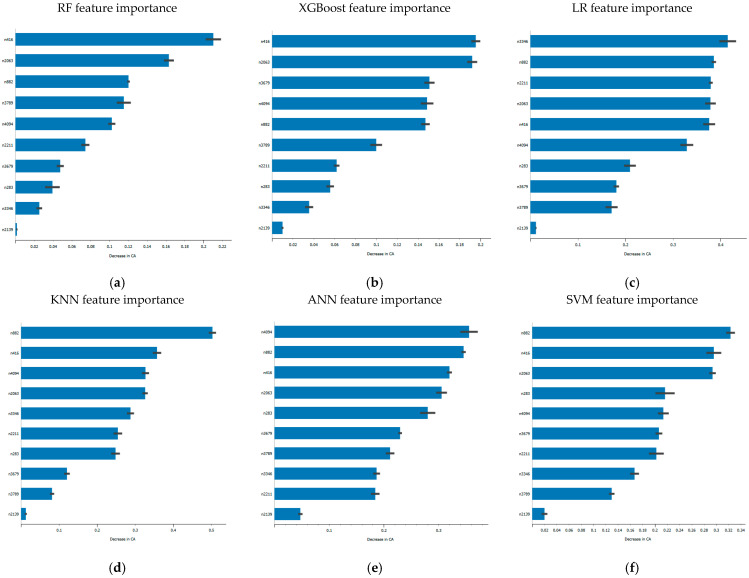
Top feature per model: (**a**) RF, (**b**) XGBoost, (**c**) LR, (**d**) KNN, (**e**) ANN, (**f**) SVM.

Figure 10 illustrates examples of the most influential features identified for each classification model on Dataset B, based on the corresponding decrease in accuracy. This dual-stage selection process not only reduces computational complexity and enhances model generalization but also significantly contributes to provide global interpretability. By systematically linking the CNN-derived features to their impact on classification outcomes, the proposed method transforms abstract deep features into transparent, tractable inputs for supervised models. This approach mitigates the inherent opacity of CNN models by enhancing the traceability of their outputs, thereby promoting greater explainability, reliability, and accountability in bearing fault diagnosis framework.

This study employs the tenfold cross-validation (CV) method [48] to evaluate the effectiveness of the proposed rolling bearing fault recognition strategy and to obtain an unbiased performance estimate of the various machine learning algorithms adopted in this work. In each fold, 90% of the data is used for training and 10% for testing, ensuring that every sample appears in the test set exactly once across all iterations. Each partition also maintains a balanced distribution of class samples to preserve fault type representativeness throughout the validation process. The overall performance is then computed as the average accuracy across the ten folds.

To rigorously prevent test–training data leakage, the data are first partitioned into folds, and for each iteration, all preprocessing, feature engineering, and model training are performed solely on the training subset of that fold. The test data remain completely unseen and isolated until the final evaluation stage in each fold. This strict separation ensures that the model does not gain access directly or indirectly to any information from the test set during training or feature selection, thereby eliminating leakage and preserving the validity of the results. Furthermore, the following metrics measurements as described in [49,50] are used to evaluate the performance of each classification algorithm.

- Classification accuracy (CA) is computed by dividing the number of correct assessments for a particular class by the total number of assessments for that class. Accuracy represents the efficiency of classification.
- Precision describes which rolling bearing fault is recognized as belonging to a particular class actually belongs to this class.
- Recall determines which rolling bearing fault is recognized correctly.
- F1-score functions as a single assessment value for classification models by computing the harmonic mean between precision and recall.
- AUC (area under the receiver-operating curve) is an interpolation between the true positive and false positive rates. It is used to analyze the performance of classification models. An AUC greater than 90% indicates an excellent classification result.
- Execution time in machine learning execution time is cumulated time in seconds used in training and testing the model.

Our experiments were carried out on a computer running Microsoft Windows 10 operating system and an Intel^®^ Core (TM) i5-11400H CPU with 16 GB memories, using Python 3.9.12. The proposed system was evaluated on two datasets: one with 4 classes and another with 10 classes, covering various bearing fault types and severity levels. To achieve the optimal results for each classifier, we designed models according to the kernel hyperparameters that illustrated in Table 5.

A detailed comparison among various classifiers including: FR, XGBoost, LR, KNN, ANN, SVM on all the performance parameters is given in Table 6. The comprehensive analysis indicates a real improvement of all the machine learning models in classifying rolling bearing faults, adopting the workflows proposed in this study. Particularly, in terms of diagnostic accuracy and computational efficiency, the Support Vector Machine (SVM) algorithm exhibited superior performance achieving classification accuracies of 100% and 99.66%, with notably reduced computational times of 0.1 s and 6.8 s on Datasets A and B, respectively.

**Figure 11:**
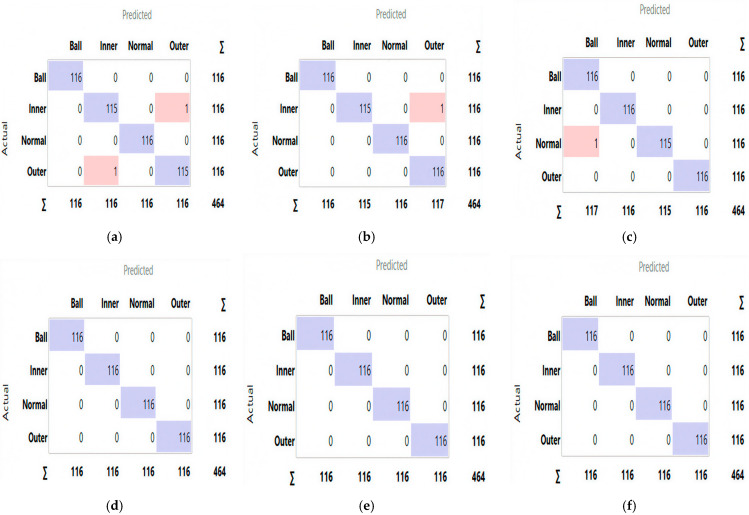
The confusion matrix of proposed models ((**a**) RF, (**b**) XGBoost, (**c**) LR, (**d**) KNN, (**e**) ANN, (**f**) SVM) with the dataset A.

**Figure 12:**
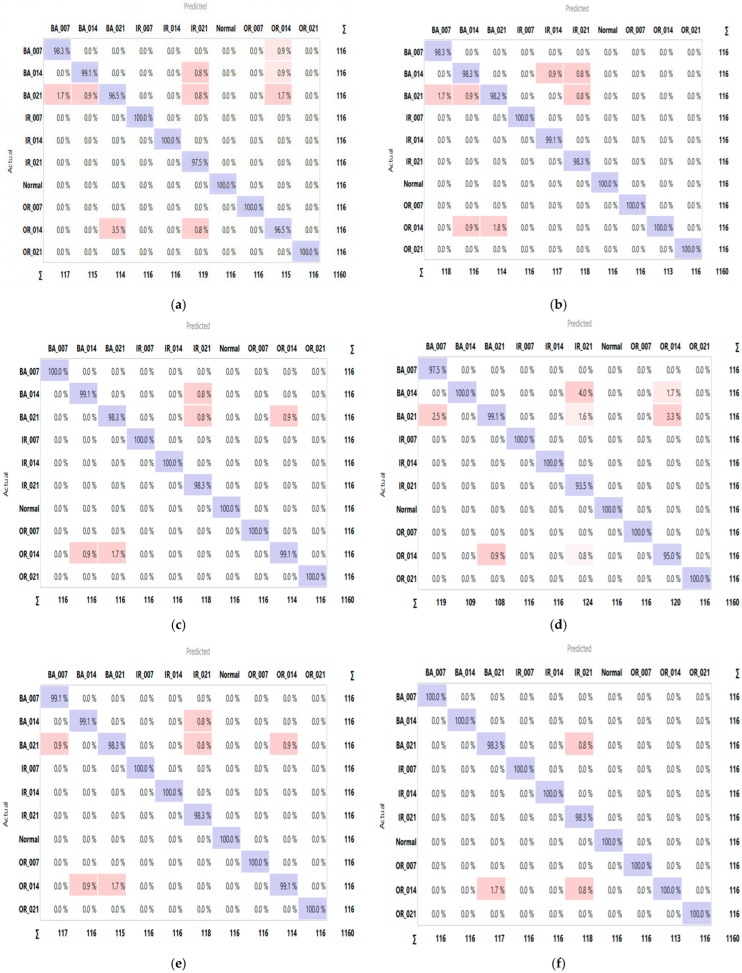
The confusion matrix of proposed models ((**a**) RF, (**b**) XGBoost, (**c**) LR, (**d**) KNN, (**e**) ANN, (**f**) SVM) with the dataset B.

**Figure 13:**
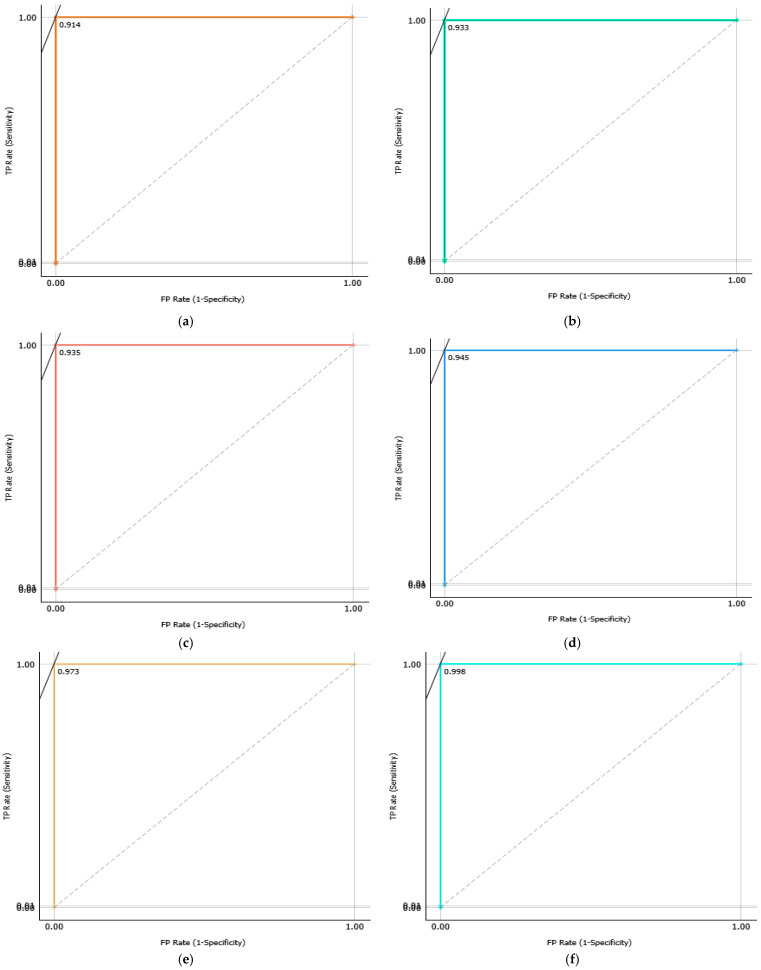
ROC curves of proposed models ((**a**) RF, (**b**) XGBoost, (**c**) LR, (**d**) KNN, (**e**) ANN, (**f**) SVM) with the dataset A.

**Figure 14:**
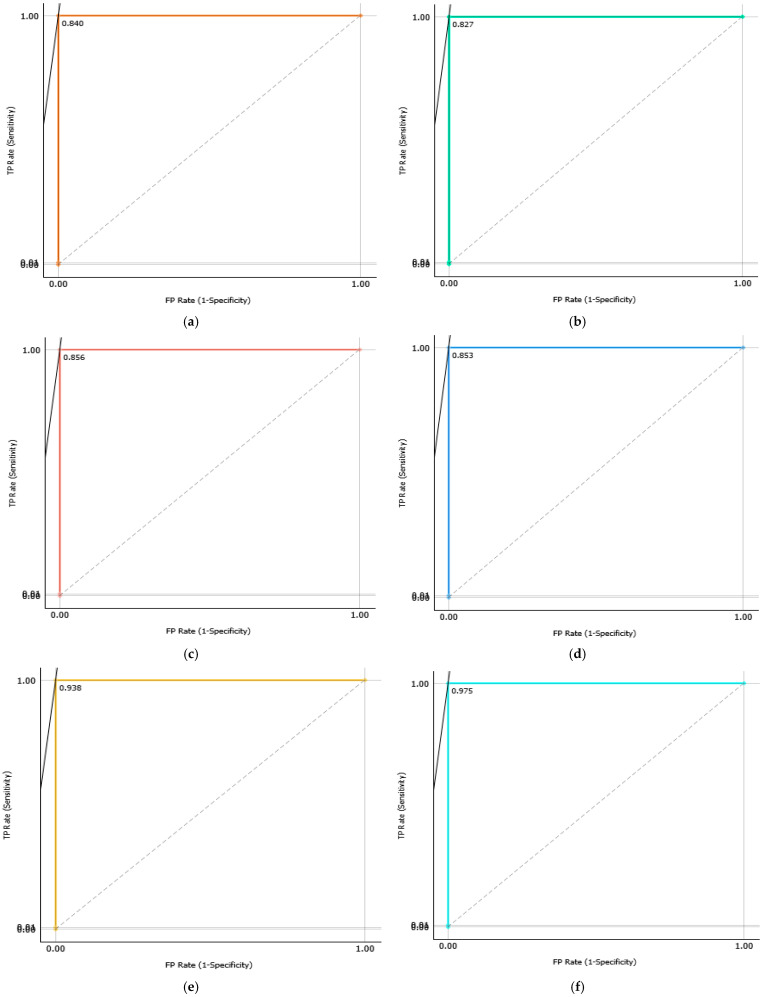
ROC curves of proposed models ((**a**) RF, (**b**) XGBoost, (**c**) LR, (**d**) KNN, (**e**) ANN, (**f**) SVM) with the dataset B.

A comparative analysis was conducted to assess the effectiveness of the proposed system with and without the dual-stage feature selection approach. As shown in Table 7, feature selection consistently improved both classification accuracy and computational efficiency across Datasets A and B. For instance, the SVM model achieved an average accuracy gain of 0.41% on Dataset B, while its computational time decreased significantly from 34 s to 6.8 s, demonstrating the ability of the dual-stage method to eliminate redundant features and enhance classifier performance. The confusion matrices (Figure 11 and Figure 12) provide a detailed view of class-wise predictions, while the ROC curves (Figure 13 and Figure 14) confirm the models’ strong discriminatory capability through high true positive rates and low false positive rates.

Importantly, these improvements extend beyond algorithmic performance to directly enhance defect identification in rolling bearings. The higher accuracies correspond to more reliable differentiation between normal conditions and specific fault types (inner race, outer race, and rolling element defects), while reduced computational time supports faster and more practical deployment in industrial settings. Thus, the proposed hybrid diagnostic framework not only surpasses traditional classifiers in numerical performance but also strengthens the quality of fault detection and enables timely, reliable diagnosis for predictive maintenance applications.

Table 8 summarizes a comparative analysis between the proposed system and a selection of recent methodologies applied to the CWRU dataset. The results demonstrate that the proposed approach significantly outperforms several existing techniques, exhibiting superior performance in both classification accuracy and computational efficiency.

Overall, these findings substantiate the effectiveness of the proposed strategy in achieving robust diagnostic performance while maintaining a lightweight and computationally efficient design.

Although the proposed framework demonstrates strong performance under controlled experimental conditions, we acknowledge that the framework relies on labeled data for supervised learning, which may limit its generalizability to unlabeled or real-world scenarios. The framework has been primarily validated on relatively well-defined fault types within a specific dataset, which may not fully capture the complexity and variability of faults occurring in diverse, real-world machinery and operational conditions. Its effectiveness may be challenged in noisy industrial environments where vibration signals are often contaminated with significant background noise and interference. Such noise can degrade the quality of the extracted features and complicate accurate fault classification. Complex mechanical systems often exhibit overlapping fault signatures and evolving degradation patterns that could reduce the robustness and generalizability of the model.

## 8. Model’s Explanation via SHAP Analysis

Building upon the global interpretability insights provided by PFI, this section introduces SHapley Additive exPlanations (SHAP) to achieve local interpretability explaining individual predictions and elucidating how specific deep features influence model outputs [51]. While PFI quantifies the overall importance of each feature by measuring its impact on model performance when permuted, SHAP offers a more granular perspective by attributing a precise contribution of each feature to a single prediction [52]. This localized interpretability is essential in high-stakes domains such as bearing fault diagnosis, where model transparency, trust, and robustness are critical for reliable decision-making [53].

SHAP is grounded in cooperative game theory and computes feature attributions based on Shapley values, the average marginal contribution of each feature across all possible feature combinations [54]. In addition to PFI which provides global rankings, SHAP explains why a model outputs a specific prediction for a given input, identifying whether each feature increases or decreases the likelihood of a particular class [51,55]. Thus, SHAP not only complements PFI but enhances interpretability by revealing the direction and magnitude of each feature’s influence at the instance level.

**Figure 15:**
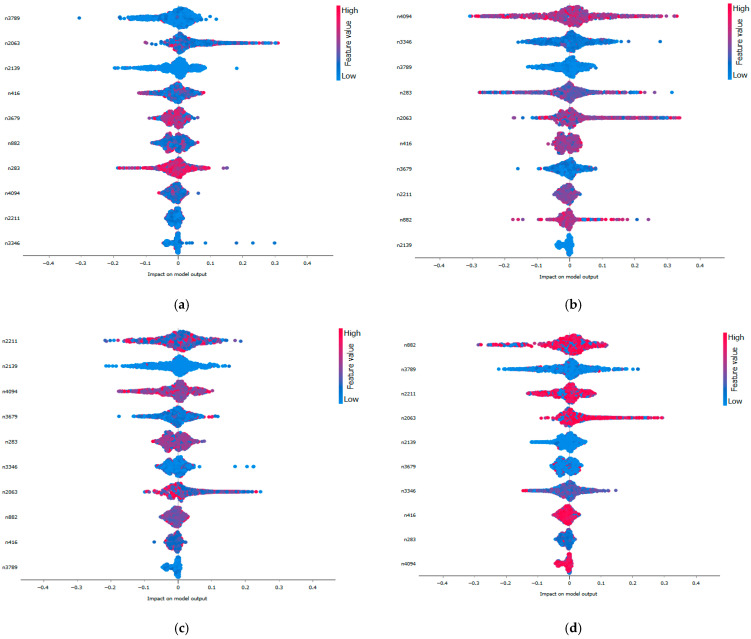

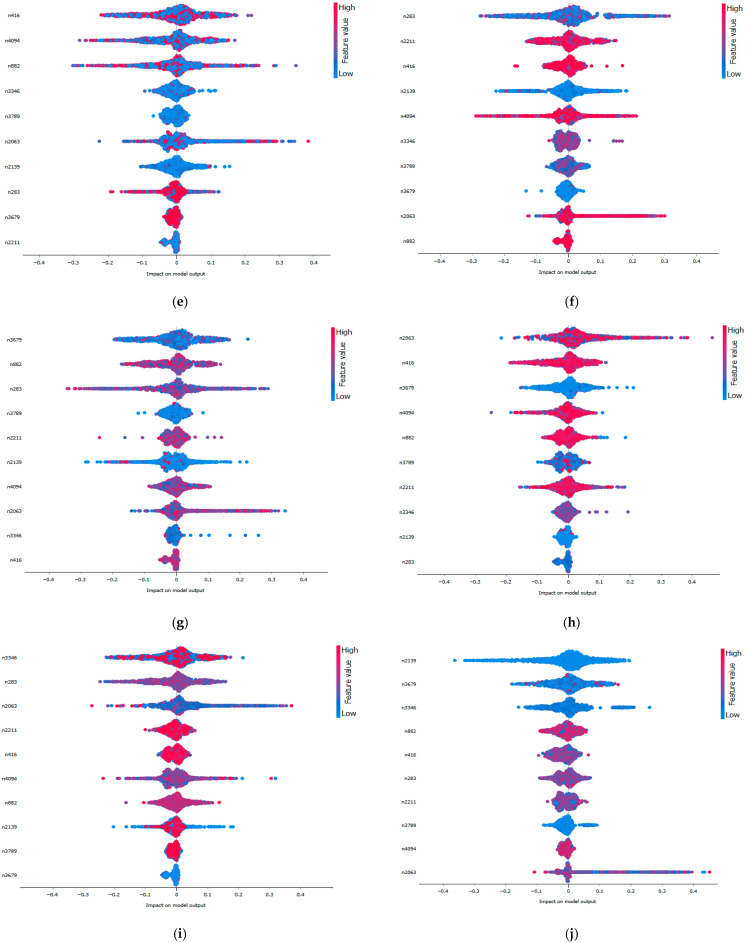
SVM Shapley values for each fault class: ((**a**) IR_007, (**b**) IR_0014, (**c**) IR_021, (**d**) OR_007 (**e**) OR_014, (**f**) OR_021 (**g**) BA_007, (**h**) BA_0014, (**i**) BA_021, (**j**) Normal) in dataset B.

Figure 15 illustrates the SHAP values obtained for the different fault classes with dataset B, using the Support Vector Machine (SVM) model as a representative example. The plots display the initial features selected through the proposed dual-stage feature selection approach arranged along the y-axis according to their impact on the model’s output. These features are ranked by their mean SHAP values, with the x-axis representing the average contribution to the prediction. A positive SHAP value for a feature indicates that its presence increases the likelihood of the corresponding fault class, while a negative SHAP value implies a shift away from it.

This visualization facilitates a clear understanding of the contribution of individual features to the model’s decision-making process. For example, a low value of feature n283 positively influences the prediction of fault classes IR_007, IR_014, and IR_021, while negatively impacting the classification of OR_007, OR_014, and OR_021. Conversely, a high value of feature n416 enhances the likelihood of predicting fault classes OR_007, OR_014, and OR_021, while exerting a negative effect on the prediction of IR_007, IR_014, and IR_021.

In this way, it becomes evident how individual features differentially affect class-specific outcomes, offering a nuanced understanding of the model’s internal logic.

Such insight reveals that certain deep features such as n283 and n416 consistently correspond to characteristic vibration behaviors of specific fault types. Features strongly linked to inner race faults capture transient, high-frequency components associated with the impulsive impacts caused by inner race defects. Conversely, features driving outer race fault predictions exhibit periodic patterns reflecting the stationary nature of outer race damage.

Thus, SHAP serves as a bridge between abstract deep features and physically interpretable fault mechanisms, offering insight into how the model internally differentiates between fault types based on the signal characteristics they produce.

This analysis can be systematically extended to all machine learning models employed in this study, allowing consistent evaluation of feature contributions across classifiers. The integration of SHAP within the proposed hybrid diagnostic framework thus provides a critical layer of interpretability, facilitating transparent, trustworthy, and actionable insights in the context of real time intelligent bearing fault diagnosis.

## 9. Conclusions

This study presented a hybrid diagnostic framework that effectively bridges the gap between high-accuracy fault detection and model interpretability in vibration-based condition monitoring of rotary machinery. By integrating deep feature extraction through VGG19-based transfer learning with a dual-stage feature selection mechanism, comprising ANOVA and PFI, the framework enables both superior diagnostic accuracy and enhanced model transparency. This combination facilitates the identification of the most discriminative CNN-derived features, providing a systematic understanding of the representations driving fault classification.

A central innovation of the proposed framework lies in its dual-stage feature selection strategy, which leverages both statistical relevance (ANOVA) and model-based importance (PFI) to systematically refine deep features for classification. This approach, to the best of our knowledge, has not been extensively explored in vibration-based fault diagnosis. Combined with SHAP-based interpretability analysis, the framework not only achieves high diagnostic accuracy but also ensures transparency in the classification process, an essential requirement for practical industrial applications.

Furthermore, SHAP analysis is employed to rigorously interpret the predictions of the supervised models, offering quantitative insight into individual feature contributions. This deepens understanding of the decision-making process and substantially improves the transparency, trustworthiness, and accountability of the overall system.

Experimental validation using the CWRU bearing dataset confirms the effectiveness of the proposed approach, achieving up to 100% classification accuracy while maintaining computational efficiency suitable for real-time applications. Overall, the framework advances the field by transforming complex deep learning outputs into interpretable, reliable insights, thereby supporting trustworthy, transparent decision-making in safety-critical industrial environments. Consequently, further investigation is necessary to adapt and optimize the methodology for practical applications involving multi-source noise and more intricate fault scenarios typical of industrial environments, where signal contamination and overlapping fault signatures may pose additional challenges. In addition, future work will aim to extend the proposed framework by incorporating multi-sensor data fusion, integrating heterogeneous sources such as acoustic emissions and thermal measurements to enhance the robustness and reliability of fault diagnosis. Moreover, the development and evaluation of computationally efficient model architectures and feature selection techniques specifically optimized for deployment on edge computing platforms will be prioritized, facilitating real-time condition monitoring within resource-constrained environments. Furthermore, efforts will be directed towards validating the generalizability of the framework across a wider variety of machinery types and fault modalities. In addition, the exploration of advanced model interpretability methods beyond SHAP, including but not limited to counterfactual explanations and causal inference techniques, is anticipated to yield deeper insights into model decision-making processes and further advance transparency in industrial prognostic systems.

## Figures and Tables

**Table 1:** IEEE distribution of common faults in electric motors.

Fault Category	Approximate Contribution (%)
Bearings faults	40–50%
Stator faults	30–40%
Rotor faults	~10%
Other faults	<10%

**Table 2:** Bearing’s technical parameters.

Technical Parameters	Value
Inner race diameter d	25.001 (mm)
Outer race diameter D	51.998 (mm)
Ball diameter B_D_	7.940 (mm)
Pitch diameter C_D_	39.04 (mm)
Ball number N	9 (mm)
Overall width B	15 (mm)
Contact angle β	0 (rad)

**Table 3:** The fault classes used from the CWRU dataset.

Faut Class	Fault Diameter (Inch)	Fault Class
Healthy status	/	Normal
Inner race	0.007	IR_007
Outer race (centered at 6:00)	0.007	OR_007
Ball	0.007	BA_007
Inner race	0.014	IR_014
Outer race (centered at 6:00)	0.014	OR_014
Ball	0.014	BA_014
Inner race	0.021	IR_021
Outer race (centered at 6:00)	0.021	OR_021
Ball	0.021	BA_021

**Table 4:** The constructed dataset.

Dataset	NB of Classes	Load (HP)	NB of Images
A	4	0, 1, 2 and 3	464
B	10		1160

**Table 5:** Hyperparameters used for classification models.

Classification Model	Hyperparameters Definition	Values
Random Forest	Number of trees	100
(RF)	Number of attributes at each split	10–15
Extreme Gradient Boosting	Booster	gptree
(XGBoost)	Learning rate	0.01–0.3
	Limit depth	7
	Number of trees	100
	Subsample	0.5
Logistic Regression	Lasso regression	Lasso (L1)
(LR)	Strength C	1
K-Nearest Neighbors	Number of neighbors	5–10
(KNN)	Function	Euclidean
Artificial Neural Networks	Number of neurons in hidden layers	100
(ANN)	Activation function	ReLu
	Optimizer	Adam
	Learning rate	0.001
Support Vector Machine	Kernel function	Polynomial
(SVM)	Kernel degree	3
	Cost C	1
	Gamma	0.1

**Table 6:** Comparative analysis of model performance metrics on Datasets A and B.

Metric Performance	Dataset	RF	XGBoost	LR	KNN	ANN	SVM
Average accuracy		99.55%	99.76%	99.78%	100%	100%	100%
Precision		99.60%	99.80%	99.80%	100%	100%	100%
Recall	A	99.55%	99.76%	99.8%	100%	100%	100%
F1-score		99.50%	99.65%	99.70%	100%	100%	100%
AUC		99.65%	99.80%	99.85%	100%	100%	100%
Average execution time (S)		1.7	0.6	0.5	0.01	2.4	0.1
Average accuracy		98.79%	99.22%	99.56%	98.51%	99.33%	99.66%
Precision	B	98.50%	99.20%	99.60%	98/50%	90.40%	99.70%
Recall		98.79%	99.22%	99.56%	98.51%	99.33%	99.66%
F1-score		98.22%	99.02%	99.49%	98.15%	99.13%	99.36%
AUC		99.11%	99.45%	99.55%	99.65%	99.81%	100%
Average execution time (S)		7.7	5.7	9.1	3.1	19	6.8

**Table 7:** Classification performances with and without dual-stage feature selection.

Model	Dataset	Without Feature Selection Approach		With Feature Selection Approach	
		Accuracy	Execution Time	Accuracy	Execution Time
RF		99.00%	4.1	99.55%	1.7
XGBoost		99.01%	2.8	99.76%	0.6
LR	A	99.11%	2.5	99.78%	0.5
KNN		99.78%	1.3	100%	0.01
ANN		99.70%	44	100%	2.4
SVM		100%	5.5	100%	0.1
RF		98.40%	15.3	98.79%	7.7
XGBoost		98.80%	17.1	99.22%	5.7
LR		99.12%	23.2	99.56%	9.1
KNN	B	98.22%	13.2	98.51%	3.1
ANN		99.10%	68.1	99.33%	19
SVM		99.25%	34	99.66%	6.8

**Table 8:** Comparative results with existing recent works on CRWU dataset.

Reference/Year	Methods Used	Execution Time (S)	Average Accuracy
[26]/(2019)	CWT-CNN-RF	99.08	114.57
[27]/(2022)	Three stage learning-		
	SVM	N/A	86.11%
	KNN	N/A	92.59%
	ANN	N/A	95.37%
[29]/(2023)	VMD-ANN	2.4	99.3%
[11]/(2023)	AE-Fine KNN	12.26	100%
Our models	Hybrid framework-		
	RF	1.7	99.55%
	XGBoost	0.6	99.76%
	LR	0.5	99.78%
	KNN	0.01	100%
	ANN	2.4	100%
	SVM	0.1	100%

## Data Availability

This study uses the publicly available CRWU dataset https://csegroups.case.edu/bearingdatacenter, accessed on 15 February 2024.

## Nomenclature

ANOVA	Analyze of Variance
PFI	Permutation Feature Importance
SHAP	SHapley Additive exPlanations
CV	Cross Validation
